# Use of an electronic Partograph: feasibility and acceptability study in Zanzibar, Tanzania

**DOI:** 10.1186/s12884-018-1760-y

**Published:** 2018-05-09

**Authors:** Lindsay Elizabeth Litwin, Christina Maly, Asma Ramadan Khamis, Cyndi Hiner, Jérémie Zoungrana, Khadija Mohamed, Mary Drake, Michael Machaku, Mustafa Njozi, Salhiya Ali Muhsin, Yusuph K. Kulindwa, Patricia P. Gomez

**Affiliations:** 1 Jhpiego, an affiliate of Johns Hopkins University, 1615 Thames Street, Baltimore, MD 21231 USA; 2 Jhpiego, an affiliate of Johns Hopkins University, Plot 72, Block 45B, Victoria Area, New Bagamoyo Rd, PO Box 9170, Dar es Salaam, Tanzania; 3 Ministry of Health Zanzibar, Box 236, Stone Town, Zanzibar Tanzania

**Keywords:** Partograph, Partogram, Maternal and newborn health, Labor and delivery, Mobile data collection, Zanzibar, Clinical decision support, Quality of care, Real-time monitoring

## Abstract

**Background:**

The ePartogram is a tablet-based application developed to improve care for women in labor by addressing documented challenges in partograph use. The application is designed to provide real-time decision support, improve data entry, and increase access to information for appropriate labor management. This study’s primary objective was to evaluate the feasibility and acceptability of ePartogram use in resource-constrained clinical settings.

**Methods:**

The ePartogram was introduced at three facilities in Zanzibar, Tanzania. Following 3 days of training, skilled birth attendants (SBAs) were observed for 2 weeks using the ePartogram to monitor laboring women. During each observed shift, data collectors used a structured observation form to document SBA comfort, confidence, and ability to use the ePartogram. Results were analyzed by shift. Short interviews, conducted with SBAs (*n* = 82) after each of their first five ePartogram-monitored labors, detected differences over time. After the observation period, in-depth interviews were conducted (*n* = 15). A thematic analysis of interview transcripts was completed.

**Results:**

Observations of 23 SBAs using the ePartogram to monitor 103 women over 84 shifts showed that the majority of SBAs (87–91%) completed each of four fundamental ePartogram tasks—registering a client, entering first and subsequent measurements, and navigating between screens—with ease or increasing ease on their first shift; this increased to 100% by the fifth shift. Nearly all SBAs (93%) demonstrated confidence and all SBAs demonstrated comfort in using the ePartogram by the fifth shift. SBAs expressed positive impressions of the ePartogram and found it efficient and easy to use, beginning with first client use. SBAs noted the helpfulness of auditory reminders (indicating that measurements were due) and visual alerts (signaling abnormal measurements). SBAs expressed confidence in their ability to interpret and act on these reminders and alerts.

**Conclusions:**

It is feasible and acceptable for SBAs to use the ePartogram to support labor management and care. With structured training and support during initial use, SBAs quickly became competent and confident in ePartogram use. Qualitative findings revealed that SBAs felt the ePartogram improved timeliness of care and supported decision-making. These findings point to the ePartogram’s potential to improve quality of care in resource-constrained labor and delivery settings.

**Electronic supplementary material:**

The online version of this article (10.1186/s12884-018-1760-y) contains supplementary material, which is available to authorized users.

## Background

Regular and timely monitoring of maternal and fetal parameters during labor is critically important to assessing maternal and fetal well-being, supporting normal labor, identifying complications and spurring clinical decision-making to address them in a timely manner. For the last four decades, the World Health Organization (WHO) has recommended that, during labor, skilled birth attendants (SBAs)^1^ use the partograph as a tool to improve documentation of intrapartum maternal and fetal measurements, identify abnormalities, and inform appropriate labor management [1]. Strengthening service delivery and improving quality of care during labor and delivery is essential for improving maternal and neonatal survival [2, 3]. While there is limited evidence that partograph use alone will improve maternal and neonatal survival [4], a multicenter WHO study demonstrated promising effects on care and labor outcomes when the partograph is implemented with a clear labor management protocol [5]. In addition, the information on the partograph can be used to reassure the woman and her family about appropriate progress in labor, or explain abnormalities and potential interventions [6].

**Fig. 1:**
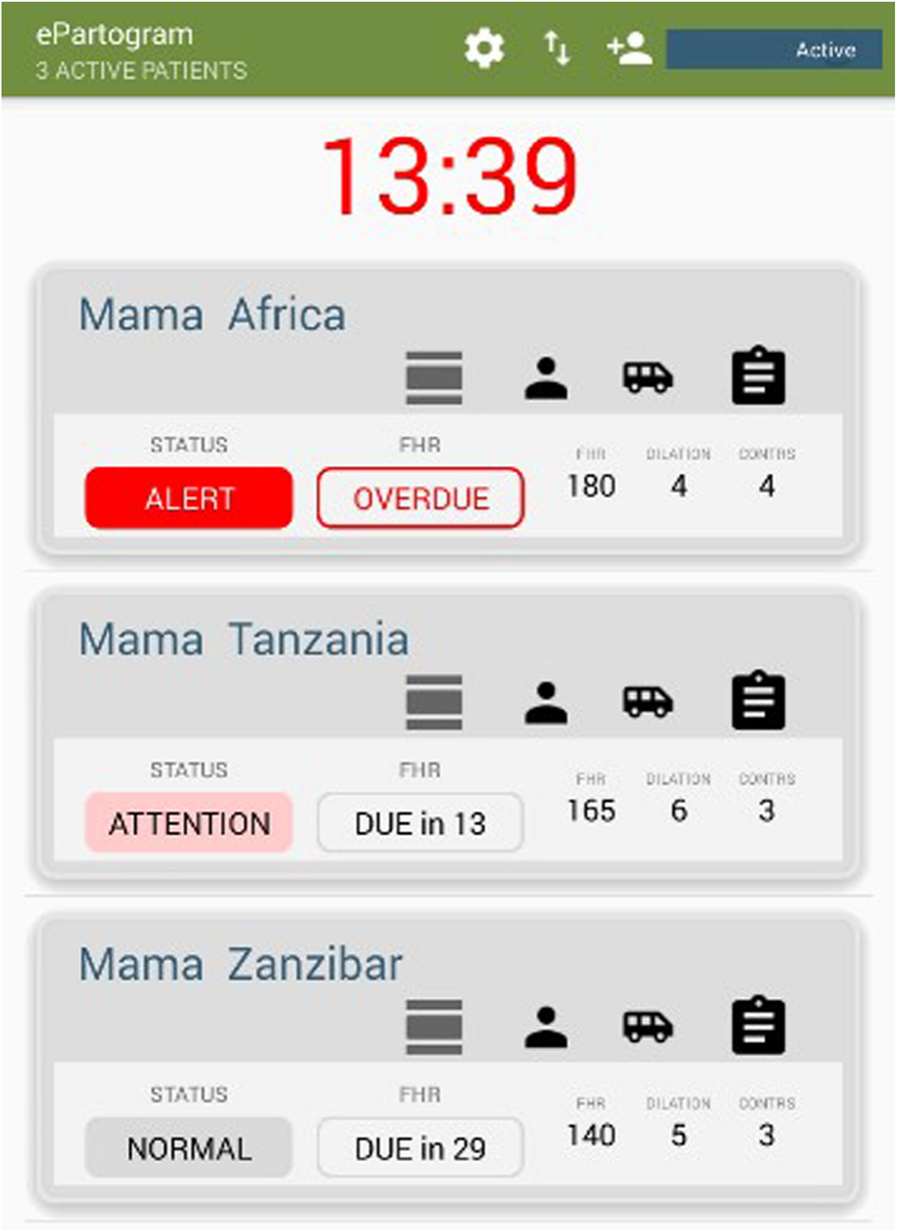
ePartogram

Routine use of the paper partograph in resource-constrained settings is low and inconsistent, with partographs often completed retrospectively for recordkeeping purposes only [7–9]. Factors contributing to suboptimal partograph use include lack of availability of partographs and labor management guidelines, insufficient knowledge, training or supportive supervision of SBAs related to partograph use, negative perceptions of the partograph and its value, and insufficient institutional commitment to partograph use [7, 10–13]. To address documented challenges, Jhpiego developed the ePartogram for use on an Android tablet, with particular attention to improving ease and efficiency of real-time documentation, increasing visibility of labor management data to nurses and supervisors to inform decision-making, and providing visual and auditory cues for timely and appropriate clinical decision-making during labor. The ePartogram was developed from 2010 to 2015 by a team of engineers, public health experts, experienced clinicians, and software developers with iterative end-user feedback, in collaboration with systems development partner D-Tree International. These stakeholders were consulted throughout the development process to inform the product specifications and design (see Additional file 1). The ePartogram is a clinical decision-support tool with algorithms that are based on WHO guidance for managing complications in pregnancy and childbirth [14]. However, because not every “clinical rule” for ePartogram use is printed in WHO documentation, WHO guidance was augmented with Jhpiego clinical expertise. Jhpiego doctors, nurses, and midwives were consulted in developing priority clinical rules that would address the leading intrapartum complications; these rules were externally validated by practicing clinicians from low-resource settings. The resulting ePartogram application includes auditory reminders that prompt SBAs to take measurements, such as obtaining fetal heart rate every 30 min, and visual alerts to flag measurements indicating the need for follow-up, for example, if the dilation measurement crosses the alert or action line (see Fig. 1).

The ePartogram is designed to increase data entry efficiency by automatically graphing data and storing all client files within the application. Overall, the ePartogram system aims to improve documentation and use of intrapartum data to inform clinical labor management decisions. It also allows every SBA in a facility to have instant access to information about all clients in active labor, which facilitates continuity of care and coordinated caseload management. The ePartogram reinforces SBA clinical expertise by supporting appropriate monitoring and care during labor. Several mobile and computer-based tools are under development with the shared vision of improving care during labor [10, 15, 16]; however, most of these products are in the early introduction phase and further rigorous research is needed [17].

This study was conducted in Zanzibar, United Republic of Tanzania, where even though 60% of births take place at a health facility [18], maternal and newborn mortality remain high. Zanzibar has a maternal mortality ratio (MMR) of 307/100,000 live births and a newborn mortality rate of 29/1000 live births [19, 20], far from the 2030 Sustainable Development Goals of 70/100,000 live births and 12/1000 live births, respectively [21]. A 2010 study on quality of maternal and newborn health services in Zanzibar found that only 58% of observed births were attended by SBAs using a partograph, and of those, only 7% of partographs had fetal heart rate, frequency and duration of contractions, and maternal pulse filled in correctly at least every 30 min [22]. These findings are similar to findings in other settings in which partograph use is low.

## Methods

### Study design and setting

A mixed methods exploratory study was designed to determine the feasibility and acceptability of ePartogram use by SBAs in resource-constrained clinical settings. Parameters of feasibility were assessed by each SBA’s ability to use the ePartogram at the point of care following a three-day training. Aspects of feasibility included ease of data entry, navigation within the application, and maintenance of the device. Acceptability was determined by evaluating SBAs’ comfort and confidence using the device while managing a client in labor.

**Fig. 2:**
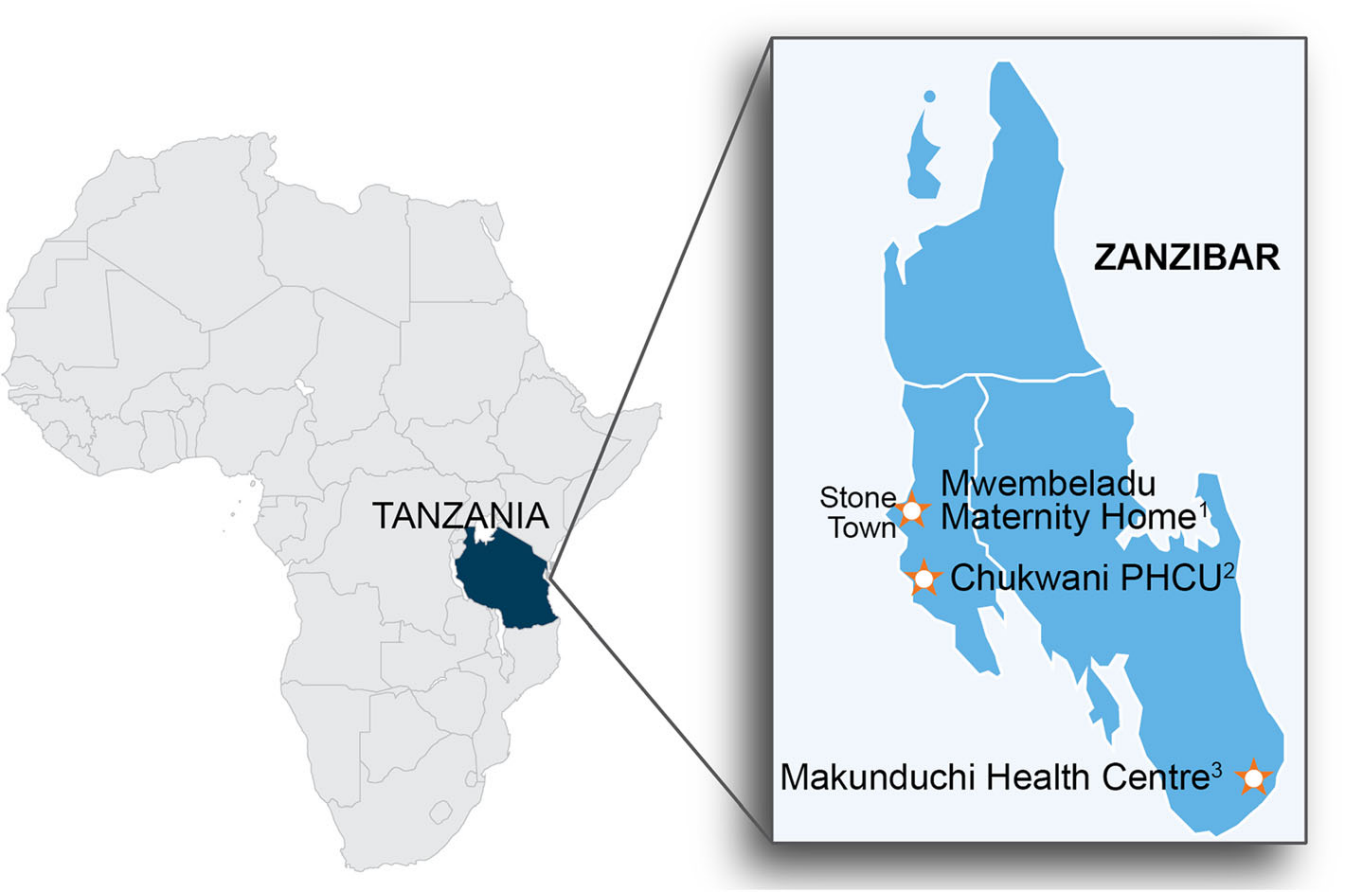
Facility profiles: ^1^Mwembeladu Maternity Home: urban, 550 births/month, 12 SBAs enrolled in study. ^2^Chukwani Primary Health Care Unit (PHCU): urban, 105 births/month, 7 SBAs enrolled in study. ^3^Makunduchi Health Centre: rural, 136 births/month, 12 SBAs enrolled in study

The study was conducted with SBAs from three purposively selected health facilities on Unguja Island in Zanzibar from April 15 through May 16, 2015. Data were collected at each facility over a two-week period. The three facilities were selected in consultation with the Zanzibar Ministry of Health (MOH) based on caseload of at least 50 births per month, 3G mobile network availability, adequate stock of paper partographs, current paper partograph use per MOH guidelines, and 24-h availability of a senior in-charge clinician to provide support to SBAs. Selected study sites included a low-volume, rural facility (Makunduchi Health Centre), a low-volume, urban facility (Chukwani Primary Health Care Unit [PHCU]), and a high-volume, urban facility (Mwembeladu Maternity Home) (see Fig. 2).

Data collection methods were direct clinical observation with a structured checklist, short semi-structured interviews, and in-depth interviews (IDIs). All tools were written in English and Kiswahili. Eight SBAs from non-study facilities were recruited as clinical observers (COs) to collect data during the study period. COs comprised nurse-midwives, clinical officers, and assistant medical officers with at least 2 years of clinical labor and delivery experience using the paper partograph. COs were required to successfully complete the same three-day training as participating SBAs and an additional four-day training focused on use of the three data collection tools, including standardization of observations, qualitative interviewing techniques, and data management, complemented with daily practice using the ePartogram.

### Study participants

All SBAs working in labor and delivery at the selected facilities were invited to participate in the study. In order to participate in the study, SBAs had to successfully complete the three-day competency-based training, which included a refresher on partograph use, instruction on how to use the ePartogram, and a discussion of the ePartogram study standard operating procedures (SOPs), and provide written informed consent. In addition, SBAs were required to pass the ePartogram skills assessment.

### Clinical observations

Clinical observations were conducted during morning, afternoon, and night shifts with a maximum of two SBAs using the ePartogram during any given shift. Each CO followed one SBA per shift and observed his/her interactions with the ePartogram using a structured observation checklist (see Additional file 2). Each SBA used the ePartogram to monitor one client at a time during the first shift, increasing the number of clients on the second and subsequent shifts according to his/her level of comfort. Any additional clients were monitored using a paper partograph. SOPs were developed to guide SBAs on client handover between shifts.

COs documented observations in four domains: ease of use, response to the ePartogram, acceptability, and caring for the tablet. During each shift, SBAs were rated on their ability to register a new client, enter the first set of clinical measurements for their client(s), enter subsequent measurements, and move between data entry screens. COs used four categories to rate each SBA’s ability to perform the tasks: accomplished with ease from the start, accomplished with increasing ease over the course of the shift, accomplished with difficulty throughout the shift, or did not accomplish. COs recorded “yes” or “no” to indicate whether or not a reminder or an alert was given during the shift; COs also selected what action(s) the provider took in response to the reminder and/or alert. During each shift, COs assessed whether or not SBAs seemed comfortable and confident using the ePartogram, selecting “yes—from start of shift,” “yes—increasingly throughout shift,” or “no—at no point during shift.” COs also recorded the following: if the tablet battery ran out during the shift, if the tablet was charged, where the SBA stored the tablet, and how it was cleaned. The structured checklist included space to document notes and any questions the SBA had about how to use the ePartogram. Quantitative observation data were entered into a database and analyzed by shift (1, 2, 3, 4, 5+) using Stata (StataCorp. 2015. Stata Statistical Software: Release 14. College Station, TX: StataCorp LP). Notes on questions raised during data collection were used by the study team to identify any additional training needs.

### SBA interviews

Each CO conducted a short semi-structured interview with the observed SBA in Kiswahili after each of the first five labors during which the SBA used the ePartogram to monitor the client. This interview obtained immediate feedback, which was recorded on a semi-structured interview guide on ePartogram use and any challenges encountered (see Additional file 3). COs took notes in either English or Kiswahili, based on their preference. After completion of direct observations, a subset of SBAs were interviewed by a CO in Kiswahili and audio recorded to gather additional insights and analysis regarding ePartogram use, including how it compared to their prior experience using a paper partograph (see Additional file 4). Convenience sampling was used to identify SBAs for IDIs, and SBAs were interviewed until saturation was reached. Interview notes captured in Kiswahili were translated into English. IDI audio-recordings were transcribed in Kiswahili, and translated into English for analysis. De-identified interview data were then analyzed by two study team members using thematic analysis techniques.

### Ethical and safety considerations

This study was approved by the Zanzibar MOH Medical Research and Ethics Committee (IRB00004514) and the Johns Hopkins University Bloomberg School of Public Health Institutional Review Board (IRB) (IRB00000758). In addition to the IRB approval, to gain community support and acceptance for the study, the study team conducted an orientation meeting with the MOH, facility in-charges, and community leaders. Clients provided oral consent to SBAs for their labors to be monitored using the ePartogram. In addition, to mitigate risks associated with potential tablet or software failure, and to adhere to MOH recordkeeping standards, COs completed a paper partograph for each labor the SBA monitored using the ePartogram. The SBA reviewed the paper partograph to ensure its accuracy; it was then retained in the client’s medical record. To ensure that SBAs managed care during labor and delivery in accordance with the normal standard of care, the ePartogram was only used when a senior clinician was on duty or on call at the facility in case of emergency. The software included a disclaimer stating, “Investigational Application: Exercise clinical judgment based on established national standards of practice.” The study coordinator also had daily debriefs with the COs, providing ongoing support and coaching as needed. Facilities were supplied with at least four Samsung Tab3 7-in. tablets to accommodate up to two SBAs using the ePartogram per shift and provide a minimum of two backup tablets in case any unanticipated technology issues arose. Tablets had screen protectors and cases. Infection prevention protocols and supplies were distributed to the facilities.^2^

## Results

COs observed 23 SBAs using the ePartogram to monitor 103 women in labor over 84 shifts in labor and delivery wards across the three sites. COs conducted a total of 82 short interviews with SBAs after having used the ePartogram to monitor their first five clients. COs also conducted 15 IDIs with SBAs after the conclusion of observations.

### Feasibility

**Fig. 3:**
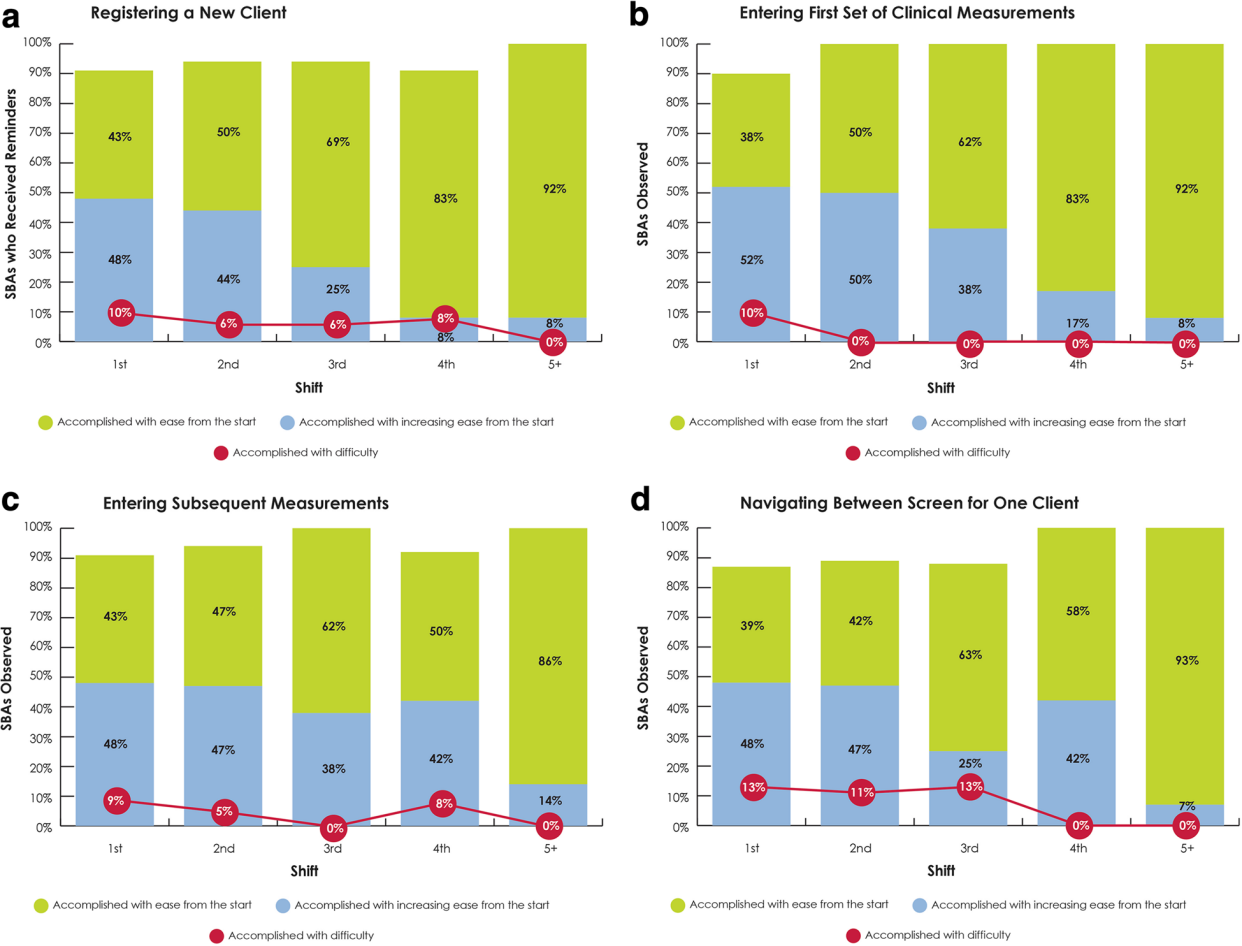
ePartogram ease of use by shift. Observed ease of use in completing tasks on the ePartogram: (**a**) registering a new client; (**b**) entering the first set of clinical measurements; (**c**) entering subsequent data; (**d**) navigating between data entry screens

Of the 31 SBAs who enrolled, 30 successfully completed the three-day training (97%) with one SBA withdrawing after not passing the skills checklist. Once they were in the clinical setting, the vast majority of SBAs were able to complete four basic tasks on the ePartogram either with ease from the beginning of the first shift or with increasing ease throughout the first shift: registering a new client in the ePartogram (43% “with ease,” 48% “with increasing ease throughout shift”), entering the first set of clinical measurements in the ePartogram (38, 52%), entering subsequent data (43, 48%), and navigating between data entry screens in the ePartogram (39, 48%) (Fig. 3). The proportion of SBAs completing these tasks with ease from the beginning of a shift steadily increased over each observed shift. By the fifth shift, no SBAs were observed having difficulty with any of the four tasks.

Based on short interviews, SBAs generally reported that they felt comfortable using the ePartogram when they first used it with a woman in labor. A couple of SBAs did report difficulty understanding where to enter maternal and fetal data the first time they used the ePartogram, but this was no longer a concern with their second client. SBAs who had used the ePartogram for two or more clients expressed that the ePartogram was increasingly easy to use and that data entry was simple and quick. During the IDIs one provider stated, “*honestly [the ePartogram] simplifies a lot during entering data [and in] follow-up about [a] patient’s labor.*” Another provider remarked how simple it was to use the application, “*Everything that you want to check on the mother is already there on the screen; what you have to do is to press the button.”*

Other aspects of feasibility included maintenance and management of the tablet by SBAs. Results showed that on their first shift, more than 80% of SBAs consistently cleaned both the tablet and tablet cover according to the SOP^3^ and that by the fifth shift this increased to 100% of observed SBAs. The tablet battery ran out three times across all shifts (81/84, 3.5%). In all other shifts the tablet battery lasted or was charged when low. During their first shift, about half of the SBAs (52%) kept the tablet in a central location or on a table, with other SBAs choosing to keep it in their pockets (26%) or on the charger (17%). By the fifth shift, the vast majority of SBAs (85%) were keeping the tablet in a central location.

In interviews, some SBAs expressed concern that the ePartogram might be challenging to use in an environment with staffing shortages or in a facility with a high client-to-provider ratio. A few SBAs raised concerns about breaking the tablet or losing data if the battery ran out, although this did not occur during the study.

### Acceptability

Acceptability was assessed through SBA interviews and by observing SBAs’ comfort and confidence as they used the ePartogram. Short interviews and IDIs revealed that SBAs had positive impressions of the ePartogram, beginning from first use with a client. After the first time using the ePartogram with a client, an SBA said, “*It is good because it reminds me of my responsibilities, it improves my self-confidence and my working prowess, and when it sounds off, I make the required decisions*.” Positive responses continued throughout the series of short interviews. After using the ePartogram with five clients, one SBA concluded, “*Truly, it is simple to use.”* During the IDIs, SBAs reiterated the positive aspects of the ePartogram as a timesaver and a tool to help organize their work. It was widely reported that the content and information contained within the ePartogram was similar to that of the paper partograph. However, the ePartogram was perceived by SBAs to be a timesaver, *“.*. *. we enter our data on the spot ‘tap, tap, tap,’ but when using the paper one, you have to take the paper and write down, at the same time taking tests. That wastes time.”*

SBAs reported that the reminders—auditory alarms indicating when a clinical measurement is due—were clear and helpful, and at times brought a greater sense of professional fulfillment, *“I was glad as it reminded me, and the mother was also happy because she saw and appreciated the good service she was given.”* In the IDIs, the SBAs expressed confidence in their abilities to interpret the alerts—visual indicators of an abnormal clinical value—and felt that they led to earlier action because “*you can easily know what is happening to the mother*. *.*. *if she is facing any risk or a problem you know how to handle the mother if [it] is to transfer her or providing her with anything of importance, you provide it to her on time*.” However, many of the SBAs expressed that the alerts reinforced their existing clinical knowledge rather than changing their decision-making process.

**Fig. 4:**
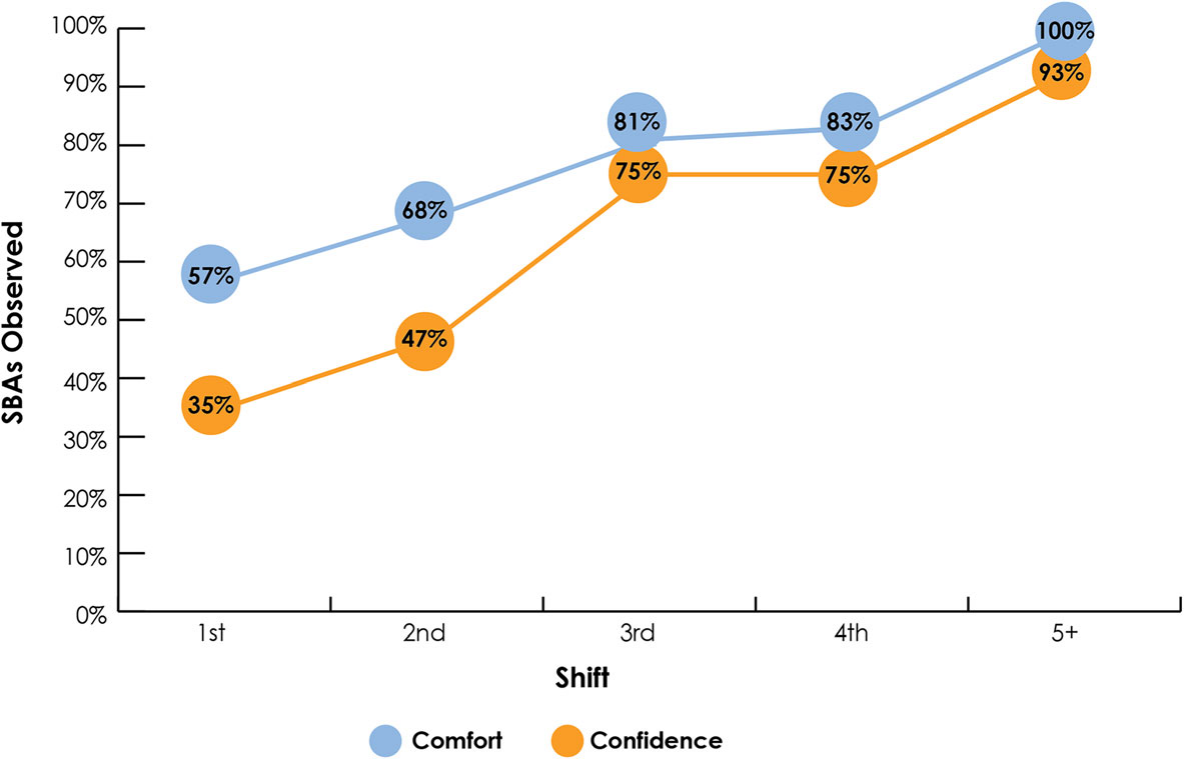
Observed SBA comfort and confidence in using the ePartogram

In addition to the qualitative findings, COs perceived that SBA confidence and comfort in using the ePartogram increased over each shift. By the fifth shift, all SBAs appeared to be comfortable using the ePartogram, and nearly all (93%) appeared confident in using it (Fig. 4).

### Potential for improved quality of care during labor

Although quality of care was not measured in this study, several findings emerged that support the ePartogram’s potential to improve the quality of care provided to women during labor in low-resource settings. Throughout short interviews and IDIs, SBAs almost universally reported that having auditory and visual reminders for which measurements were due—or were overdue—increased the frequency with which the mother was checked, improving timeliness of care. “*They have a very high value because when using a paper I will continue with other tasks, but with reminders when they ring you know that now the client needs to be tested something so you go.*” Another SBA expressed that reminders *“.*. *. will wake you up to take action to the mother.”* Several SBAs also noted that ePartogram reminders and alerts added value to paper partograph functionality.

**Fig. 5:**
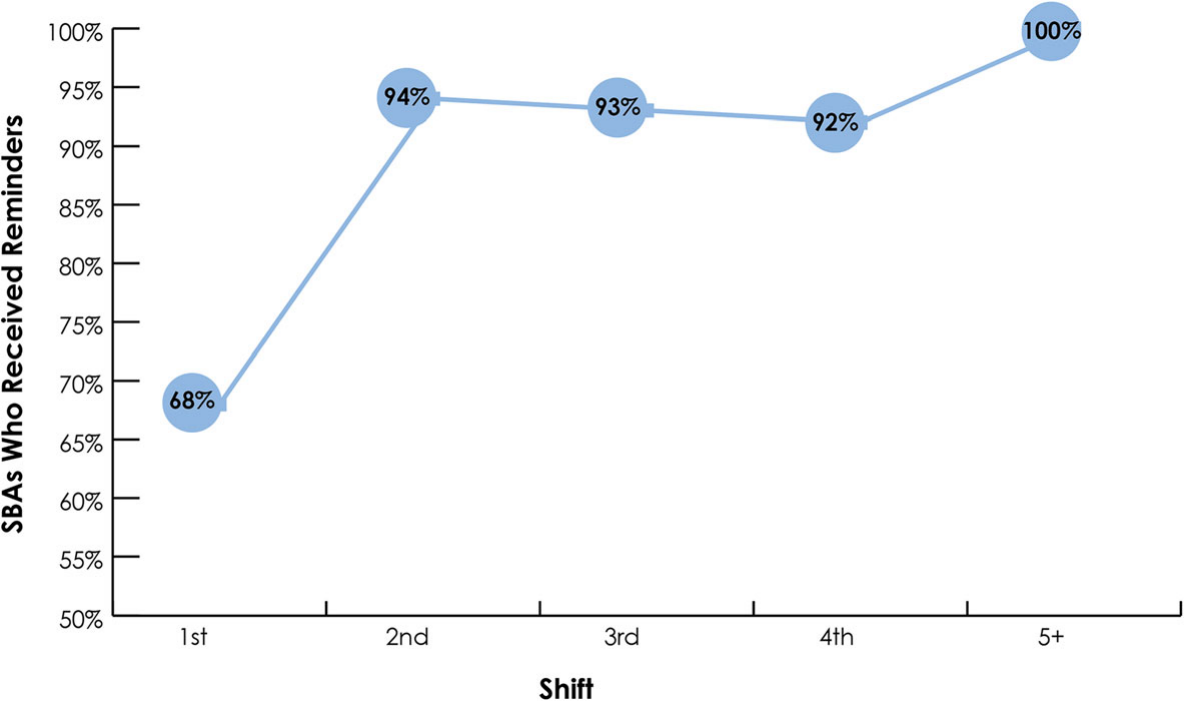
SBAs taking a measurement after a reminder

During 95% of observed shifts, a reminder sounded, indicating that measurements were due to be taken. Observation data also showed that these reminders spurred SBAs to action, primarily to take measurements. By the fifth observed shift, all SBAs took measurements in response to reminders (Fig. 5). During the first three shifts of ePartogram use, in three instances, an SBA was observed ignoring the reminder, and three SBAs asked for clarification from the CO about an alert; by the fourth shift, this behavior was no longer observed.

SBAs also reported that the ePartogram prompted them to make decisions and take actions they would not otherwise have taken, such as calling a senior clinician. One SBA noted that “*the screens*. *.*. *helped me make clinical decisions because they indicate[d] what is not normal and what is normal.”* One SBA felt that “*in general it helps us very much and empowers us to know what to do to the mother.*” Another SBA said, “*I felt good when I heard the reminders. They jolted me into making decisions, and they helped me a lot in my decisions.”*

At least one visual alert was given during 70% of the shifts observed during the study. SBA responses to alerts are more difficult to interpret because an alert indicates an abnormal clinical measurement for which a decision *may* need to be made, and this decision *may or may not* include taking action. In interviews, however, SBAs generally expressed appreciation for the alarms, “*You attend a patient according to what is indicated on the screen on who to give priority.”*

SBAs cited many additional benefits of using the ePartogram versus the paper partograph. Some felt that having all of their clients’ information in one place facilitated recordkeeping, making it easier to organize patient records and ensure care for the correct woman. Using the ePartogram was also helpful during client handover because SBAs could easily update an incoming colleague regarding a client’s status: “*It is simple in every aspect, knowing the patient and knowing what should be done with the patient*. *.*. *.”*

Although no specific questions about respectful maternity care were asked during short interviews or IDIs, one SBA noted that the ePartogram *“.*. *. brought me closer to the mother because when it rings I have to go and check the mother, so you get close to the mother and you get to know her progress.”* Several other SBAs expressed similar sentiments. One SBA said that “*the reminders were helpful and kept you in touch with the patient.”* Another felt that *“it has a very high value because you serve [the client] in a right way, after half an hour I do this, after four hours I should do that so she gets her rights.”* Yet another SBA said, *“I felt good because the client got the services she deserved.”*

## Discussion

This mixed methods exploratory study was designed to determine the feasibility and acceptability of ePartogram use by SBAs in low-resource clinical settings in Zanzibar, Tanzania. The study demonstrated both the feasibility and acceptability of ePartogram use among SBAs in these settings. With structured training and support during initial use, SBAs were quickly able to use the ePartogram with increasing ease, comfort, and confidence beginning with their first observed shift. Based on SBA feedback, we believe this application has the potential to improve quality of care in terms of timeliness of documentation and clinical action, continuity of care, and experience of care.

### Feasibility and acceptability

The proliferation of smartphone usage in Tanzania—where 73% of adults own a mobile phone and use it mainly for texting, taking pictures, and mobile banking—underpins a movement toward use of mobile applications to support clinical care in low-resource settings [23–25]. Furthermore, use of digital technologies may contribute to overall satisfaction and motivate user to adopt new tools [26]. It follows, then, that the majority of clinicians in countries like Tanzania have also adopted and rely on at least one form of new, constantly evolving mobile technology. Given the increasing number of mobile health applications, it is important to use best practices for structured development and testing of these technologies to ensure that they meet the needs of intended users. Although electronic decision-support tools have not yet been widely adopted at scale in low- and middle-income countries [27], further expansion of evidence-based applications is likely to occur in the near future.

Health care workers’ perception of an application’s ease of use is a key indicator for eventual adoption of the mHealth intervention [28, 29], and feasibility studies are an integral step in implementing mHealth solutions [30]. Thus, this study aimed to validate the feasibility and ease with which SBAs used the ePartogram during clinical practice. SBAs were able to use the ePartogram after a short training, and more than 85% were able to perform basic ePartogram tasks without significant difficulties during their first clinical shift. Use of interactive, case-based facilitation during training may have contributed to knowledge retention and application during real-life ePartogram use [31]. Observation and qualitative data show that SBA skills, comfort, and confidence in using the ePartogram increased markedly as providers continued to use the tool during a series of clinical shifts.

As new technology is introduced into health facilities, it is necessary to assess the factors that encourage integration into routine client care. One framework outlines three factors to be considered: motivators (extrinsic and intrinsic), choice of clinicians, and empowerment of clinicians. In our study, provider motivation was linked to extrinsic sources (such as special training and acquisition of new skills) and to intrinsic sources that measured ePartogram acceptability (such as positive perception of usefulness related to job performance and productivity, and perceived ease of use) [32]. End users need to be empowered and included in all processes as new technology is developed, adapted, adopted, and scaled up [33–35]. SBAs’ views were captured during interviews and the COs recorded any questions the SBAs asked while using the ePartogram. These inputs informed development of the next ePartogram version.

Results show that the majority of SBAs found the ePartogram to be useful and acceptable within their clinical context and further expressed their desire to integrate it fully into the care of their clients, recommending it for use in all facilities in Zanzibar.

### Quality of care

WHO defines quality of care as “the extent to which health care services provided to individuals and patient populations improve desired health outcomes. .. health care needs to be safe, effective, timely, efficient, equitable, and people-centered” [36, 37]. WHO’s quality of care framework encompasses the provision of care (use of evidence-based practices) and the experience of care (communication with and support of the woman and her family) [5]. This study shows that SBAs addressed both of these parameters as they monitored labor with the ePartogram. SBAs perceived that ePartogram reminders led them to obtain maternal and fetal measurements more regularly, and this was corroborated through direct observation, which enabled them to identify abnormalities more rapidly. The prioritized client listings and visual alerts also helped SBAs organize their work and give priority to women who potentially faced complications, and the reminders and alerts led SBAs to consider taking actions they might not have when using a paper partograph, important factors in ensuring quality of care during labor. WHO continues to place emphasis on partograph use, while simultaneously embarking on development of a simplified labor monitoring tool [10, 38], which like the ePartogram aims to improve the timeliness of measurements and the effectiveness of decision-making during labor. Likewise, other studies in East Africa corroborate the perceived improvement in care amongst providers using new mobile technologies [39, 40].

SBAs also stated that the ePartogram enhanced communication with women in labor. This statement is contrary to evidence reported by providers elsewhere that electronic health records (EHRs) diminish communication with clients [41] or have no effect on communication [42]. SBAs conversed more frequently with their clients and had more information to relay to them about their labors. SBAs reported satisfaction that they had established a relationship with their clients and that their clients appreciated their efforts.

### Strengths and limitations

The mixed methods observational study design introduced a structured process to ascertain the feasibility and acceptability of ePartogram use in labor wards. To minimize disruption in the labor ward, only two COs, per shift, could observe SBA ePartogram use. Thus, in the busier labor wards, not all SBAs on duty used the ePartogram. Given the study design, it was not possible to ascertain if acceptability and usability would remain as positive if fully scaled up in high-volume labor wards, particularly in busy facilities with staff shortages. The novelty of the ePartogram and the shortness of the data collection period may have also influenced the findings. Pragmatic considerations determined the timing of the study, but due to normal monthly variations, fewer labors were managed during the study period than expected. Despite these limitations, both quantitative and qualitative data were collected to validate findings and assess multiple components of feasibility and acceptability. Sampling from a resource-constrained health system, including both low- and high-volume facilities in rural and urban Zanzibar may improve generalizability of results.

### Further research needed

As the field of mHealth continues to expand, more rigorous evidence is needed to demonstrate whether such technologies improve outcomes [29, 43]. Specifically regarding the ePartogram, further research is needed to determine how it influences clinical decision-making during labor. A comparative research study is underway in 12 facilities in Kenya to compare the ePartogram with the paper partograph. Key comparative measurements include the timeliness and frequency of documented maternal and fetal measurements, adherence to standard of care to maintain normal labor, timeliness and appropriateness of clinical interventions for complications, and ultimately, if ePartogram use improves maternal and newborn outcomes as compared to the paper partograph. Given that provider workload and lack of equipment may persist as barriers to high-quality care on the day of birth, research is needed to evaluate provider motivation, practical scale-up, cost-effectiveness of sustained ePartogram use, as well as its effectiveness from a health systems perspective.

## Conclusion

Introduction of electronic decision-support tools is a relatively new domain that may have the potential to improve health service delivery in resource-constrained settings. This study demonstrates that it is feasible to introduce an electronic decision-support tool for SBAs in resource-constrained labor and delivery settings. SBAs rapidly learned how to use the ePartogram to monitor maternal and fetal well-being during labor. They also indicated that the ePartogram was easy to use and acceptable, and that it may improve the quality of services provided in labor wards in Zanzibar. Additional implementation research will need to assess the effects of ePartogram use on labor management practices and maternal and neonatal outcomes if implemented at scale.

## Additional files

Additional file 1: ePartogram Development Process. Detailed information on the design process to develop the ePartogram from 2012 to 2015 (DOCX 22 kb)

Additional file 2: Observation Form: Data Collection Tool 1. Bilingual primary (Swahili/English) data collection tool that trained study clinical observers used to assess how skilled birth attendants used the ePartogram (DOCX 46 kb)

Additional file 3: Short Interview Guide: Data Collection Tool 2. Bilingual primary data collection tool that trained study clinical observers used to interview skilled birth attendants after they first five clinical shifts using the ePartogram (DOCX 31 kb)

Additional file 4: In-Depth Interview Guide: Data Collection Tool 3. Bilingual primary data collection tool that trained study clinical observers used to interview skilled birth attendants at the end of the study period (DOCX 34 kb)

## Abbreviations

CO: Clinical observer
IDI: In-depth interview
IRB: Institutional Review Board
MMR: Maternal mortality ratio
MOH: Ministry of Health
PHCU: Primary Health Care Unit
SBA: Skilled birth attendant
SOP: Standard operating procedure
WHO: World Health Organization

## Footnotes

1 According to the WHO definition, a skilled birth attendant is a doctor, nurse, or midwife who is “trained to proficiency in the skills needed to manage normal (uncomplicated) pregnancies, childbirth, and the immediate postnatal period, and in the identification, management, and referral of complications in women and newborns.”

2 Clean tablet cover with gauze moistened with 0.1% chlorine solution. Clean screen with a cotton ball and solution of 1:1 70% isopropyl alcohol and distilled water. The tablet cover and screen should be cleaned after each shift and as deemed necessary by the SBA

3 SOP for cleaning the tablet cover: Use gauze moistened with 0.1% chlorine solution. SOP for cleaning the screen: Use a cotton ball with a solution of 1:1 70% isopropyl alcohol and distilled water

## References

[1] Hofmeyr GJ, Haws RA, Bergström S, Lee AC, Okong P, Darmstadt GL (2009). Obstetric care in low-resource settings: what, who, and how to overcome challenges to scale up?. Int J Gynaecol Obstet.

[2] Lawn JE, Kinney M, Lee AC, Chopra M, Donnay F, Paul VK (2009). Reducing intrapartum-related deaths and disability: can the health system deliver?. Int J Gynaecol Obstet.

[3] Lawn JE, Lee AC, Kinney M, Sibley L, Carlo WA, Paul VK (2009). Two million intrapartum-related stillbirths and neonatal deaths: where, why, and what can be done?. Int J Gynaecol Obstet.

[4] Lavendar T, Hart A, Smyth RM (2009). Effect of partogram use on outcomes for women in spontaneous labour at term. Cochrane Database Syst Rev.

[5] Kwast BE, Lennox CE, Farley TMM (1994). World Health Organization partograph in management of labour. World Health Organization maternal health and safe motherhood Programme. Lancet.

[6] World Health Organization (WHO). Standards for improving quality of maternal and newborn care in health facilities*.* Geneva: WHO; 2016.

[7] Maternal and Child Health Integrated Program (MCHIP) (2014). ICM congress 2014 partograph side meeting. Prague; 5 June 2014.

[8] Windrim R, Seaward PG, Hodnett E, Akoury H, Kingdom J, Salenieks ME (2007). A randomized controlled trial of a bedside partogram in the active management of primiparous labour. J Obstet Gynaecol Can.

[9] Kagema F, Ricca J, Rawlins B, Rosen H, Mukhwana W, Lynam P (2011). Quality of care for prevention and management of common maternal and newborn complications: findings from a national health facility survey in Kenya.

[10] Souza JP, Oladapo OT, Bohren MA, Mugerwa K, Fawole B, Moscovici L (2015). The development of a simplified, effective, labour monitoring-to-action (SELMA) tool for better outcomes in labour difficulty (BOLD): study protocol. Reprod Health.

[11] Mathibe-Neke JM, Lebeko FL, Motupa B (2013). The partograph: a labour management tool or a midwifery record?. Int J Nurs Midwifery.

[12] Ollerhead E, Osrin D (2014). Barriers to and incentives for achieving partograph use in obstetric practice in low- and middle-income countries: a systematic review. BMC Pregnancy Childbirth.

[13] Mathai M (2009). The partograph for the prevention of obstructed labor. Clin Obstet Gynecol.

[14] World Health Organization (WHO) (2000). Integrated management of pregnancy and childbirth. Managing complications in pregnancy and childbirth: a guide for midwives and doctors.

[15] Schweers J, Khalid M, Underwood H (2016). mLabor: design and evaluation of a mobile partograph and labor ward management application. Procedia Eng.

[16] Merck for mothers website. Transformation through digital innovations*.* http://merckformothers.com/our-work/equipping-providers.html. Accessed 4 Jan 2017.

[17] Balikuddembe MS, Tumwesigye NM, Wakholi PK (2017). Computerized childbirth monitoring tools for health care providers managing labor: a scoping review. JMIR Medical Informatics.

[18] Ministry of Health, Community Development, Gender, Elderly and Children (MOHCDGEC), [Tanzania Mainland], Ministry of Health (MOH) [Zanzibar], National Bureau of Statistics (NBS), Office of the Chief Government Statistician (OCGS), and ICF International (2016). Tanzania demographic and health survey and malaria indicator survey (TDHS-MIS) 2015–16.

[19] National Bureau of Statistics, Ministry of Finance, and Office of Chief Government Statistician, Ministry of State. Mortality and health. Dar es Salaam, Tanzania: Ministries of Finance and State; 2015. http://www.nbs.go.tz/nbs/takwimu/census2012/Mortality_and_Health_Monograph.pdf. Accessed 6 Jan 2017.

[20] National Bureau of Statistics (NBS) [Tanzania] and ICF Macro. Tanzania demographic and health survey 2010*.* Dar es Salaam, Tanzania: NBS and ICF Macro; 2011. http://dhsprogram.com/pubs/pdf/FR243/FR243%5B24June2011%5D.pdf. Accessed 6 Jan 2017.

[21] United Nations. Sustainable development goals: 17 goals to transform our world. http://www.un.org/sustainabledevelopment/health/. Accessed 4 Jan 2017.

[22] Plotkin M, Makene CL, Khamis AR, Currie S, Tibaijuka G, Lacoste M, et al. Quality of maternal and newborn health services in Zanzibar, 2010. Findings from selected health facilities in Unguja and Pemba. Baltimore: Jhpiego. p. 2012.

[23] Pew Research Center (2015). Cell phones in Africa: communication lifeline. Texting most common activity, but mobile money popular in several countries.

[24] Labrique AB, Vasudevan L, Kochi E, Fabricant R, Mehl G (2013). mHealth innovations as health system strengthening tools: 12 common applications and a visual framework. Glob Health Sci Pract.

[25] Lund S, Boas IM, Bedesa T, Fekede W, Nielsen HS, Sorensen BL (2016). Association between the safe delivery app and quality of care and perinatal survival in Ethiopia: a randomized clinical trial. JAMA Pediatr.

[26] BCS (2010). The infomational dividend: why IT makes you ‘happier’.

[27] World Health Organization (WHO) (2011). mHealth: new horizons for health through mobile technologies.

[28] Gagnon MP, Ngangue P, Payne-Gagnon J, Desmartis M (2016). M-health adoption by healthcare professionals: a systematic review. J Am Med Inform Assoc.

[29] Baig MM, GholamHosseini H, Connolly MJ (2015). Mobile healthcare applications: system design review, critical issues and challenges. Australas Phys Eng Sci Med.

[30] Sondaal SF, Browne JL, Amoakoh-Coleman M, Borgstein A, Miltenburg AS, Verwijs M (2016). Assessing the effect of mHealth interventions in improving maternal and neonatal care in low- and middle-income countries: a systematic review. PLoS One.

[31] Bluestone J, Johnson P, Fullerton J, Carr C, Alderman J, BonTempo J (2013). Effective in-service training design and delivery: evidence from an integrative literature review. Human Resour Health.

[32] Torres B. 3 factors that affect clinician buy-in. Reflexion Health. 2015; http://reflexionhealth.com/blog/2016/2/19/3-factors-that-affect-clinician-buy-in. Accessed 3 Dec 2016

[33] Legris P, Ingham J, Collerette P (2003). Why do people use information technology? A critical review of the technology acceptance model. Inf Manag.

[34] Vélez O, Okyere PB, Kanter AS, Bakken S (2014). A usability study of a mobile health application for rural Ghanaian midwives. J Midwifery Womens Health.

[35] Campbell JI, Aturinda I, Mwesigwa E, Burns B, Santorino D, Haberer JE (2017). The technology acceptance model for resource-limited settings (TAM-RLS): a novel framework for mobile health interventions targeted to low-literacy end-users in resource-limited settings. AIDS and Behav.

[36] Tunçalp Ö, Were WM, MacLennan C, Oladapo OT, Gülmezoglu AM, Bahl R (2015). Quality of care for pregnant women and newborns—the WHO vision. BJOG.

[37] Koblinski M, Moyer CA, Calvert C, Campbell J, Campbell OM, Feigl AB (2016). Quality maternity care for every woman, everywhere: a call to action. Lancet.

[38] Oladapo OT, Souza JP, Bohren MA, Tunçalp Ö, Vogel JP, Fawole B (2015). WHO better outcomes in labour difficulty (BOLD) project: innovating to improve quality of care around the time of childbirth. Reprod Health.

[39] Mitchell M, Getchell M, Nkaka M, Msellemu D, Van Esch J, Hedt-Gauthier B (2012). Perceived improvement in integrated management of childhood illness implementation through use of mobile technology: qualitative evience from a pilot study in Tanzania. J Health Commun.

[40] Jones CO, Wasunna B, Sudoi R, Githinji S, Snow RW, Zurovac D (2012). “Even if you know everything you can forget”: health worker perceptions of mobile phone text-messaging to improve malaria case-management in Kenya. PLoS One.

[41] Rathert C, Mittler JN, Banerjee S, McDaniel J. Patient-centered communication in the era of electronic health records: what does the evidence say? Patient Educ Couns. 2016; 10.1016/j.pec.2016.07.031. [Epub ahead of print] 10.1016/j.pec.2016.07.031 27477917

[42] Alkureishi MA, Lee WW, Lyons M, Press VG, Imam S, Nkansah-Amankra A (2016). Impact of electronic medical record use on the patient-doctor relationship and communication: a systematic review. J Gen Intern Med.

[43] Tomlinson M, Rotheram-Borus MJ, Swartz L, Tsai AC (2013). Scaling up mHealth: where is the evidence?. PLoS Med.

