# Supplementary material for: Use of an electronic Partograph: feasibility and acceptability study in Zanzibar, Tanzania
Source: BMC Pregnancy Childbirth. 2018 May 9;18:147. doi: 10.1186/s12884-018-1760-y (PMC5944152; doi:10.1186/s12884-018-1760-y)
Supplement: Supplementary file 1 — ePartogram Development Process. Detailed information on the design process to develop the ePartogram from 2012 to 2015 (DOCX 22 kb) [file 12884_2018_1760_MOESM1_ESM.docx]

Supplemental Material – ePartogram development process (File #1)
