# Supplementary material for: Use of an electronic Partograph: feasibility and acceptability study in Zanzibar, Tanzania
Source: BMC Pregnancy Childbirth. 2018 May 9;18:147. doi: 10.1186/s12884-018-1760-y (PMC5944152; doi:10.1186/s12884-018-1760-y)
Supplement: Supplementary file 2 — Observation Form: Data Collection Tool 1. Bilingual primary (Swahili/English) data collection tool that trained study clinical observers used to assess how skilled birth attendants used the ePartogram (DOCX 46 kb) [file 12884_2018_1760_MOESM2_ESM.docx]

**FOMU YA UANGALIZI / OBSERVATION FORM**

**Data Collection Tool 1**

**Study Title:** Feasibility of ePartogram Use in Zanzibar

**Principal Investigator:** Patricia Gomez

**IRB No.:**  6146

**Version No./Date**: v2 / April 22, 2015

| SBA study ID |  |  |  |  | Date | 2015 |  |  |  |  |
| --- | --- | --- | --- | --- | --- | --- | --- | --- | --- | --- |
|  |  |  |  |  |  | Year | Month | | Day | |
| CO Name |  | | | | | | | | | |

**MAELEKEZO** / INSTRUCTIONS:

- **Tafadhali kamilisha fomu kwa Kiswahili au Kiingereza si kwa lugha zote.**

Please complete form in either the English OR Swahili (not both).

- **Kamilisha tu sehemu ya “ maelezo” endapo kuna taarifa unayohisi ni muhimu kwa wafanyakazi wa utafiti kujua , ili kuboresha mafunzo au kwa utendaji kazi wa ePartogramu**

Only complete the “Notes” sections if there is information you feel is critical for study staff to know in order to improve training for, or functionality of, ePartogram

| **A** | **Urahisi wa kutumia ePartogram / Ease of using the ePartogram** | |
| --- | --- | --- |
| 1 | Kutengeneza mteja mpya (🗹moja)   - Imekamilika kwa urahisi tangu mwanzo - Imeendelea kuwa rahisi kukamilika kadiri muda wa kazi ulivyooenda - Imekamilika kwa shida kipindi chote cha zamu - Haikukamilika | Creating new client (🗹 one)   - Accomplished with ease from the start - Accomplished with increasing ease over course of shift - Accomplished with difficulty throughout the shift - Did not accomplish |
|  | **Maelezo / Notes** | |
| 2 | Kuingiza vipimo vya awali (🗹moja)   - Imekamilika kwa urahisi tangu mwanzo - Imeendelea kuwa rahisi kukamilika kadiri muda wa kazi ulivyooenda - Imekamilika kwa shida kipindi chote cha zamu - Haikukamilika | Entering first set of measurements (🗹 one)   - Accomplished with ease from the start - Accomplished with increasing ease over course of shift - Accomplished with difficulty throughout the shift - Did not accomplish |
|  | **Maelezo / Notes** | |
| 3 | Kuingiza vipimo vingine (🗹moja)   - Imekamilika kwa urahisi tangu mwanzo - Imeendelea kuwa rahisi kukamilika kadiri muda wa kazi ulivyooenda - Imekamilika kwa shida kipindi chote cha zamu - Haikukamilika | Entering subsequent measurements (🗹 one)   - Accomplished with ease from the start - Accomplished with increasing ease over course of shift - Accomplished with difficulty throughout the shift - Did not accomplish |
|  | **Maelezo / Notes** | |
| 4 | Kuhama toka mteja mmoja kwenda mwingine (🗹moja)   - Imekamilika kwa urahisi tangu mwanzo - Imeendelea kuwa rahisi kukamilika kadiri muda wa kazi ulivyooenda - Imekamilika kwa shida kipindi chote cha zamu - Haikukamilika - N/A kwa mteja mmoja (nenda swali #6) | Toggling between clients  (🗹 one)   - Accomplished with ease from the start - Accomplished with increasing ease over course of shift - Accomplished with difficulty throughout the shift - Did not accomplish - N/A – only one client (skip to Q6) |
|  | **Maelezo / Notes** | |
| 5 | Namna ya kuchagua mteja unayemtaka (🗹moja)   - Imekamilika kwa urahisi tangu mwanzo - Imeendelea kuwa rahisi kukamilika kadiri muda wa kazi ulivyooenda - Imekamilika kwa shida kipindi chote cha zamu - Haikukamilika - N/A kwa mteja mmoja(nenda swali #6) | Identifying desired client  (🗹 one)   - Accomplished with ease from the start - Accomplished with increasing ease over course of shift - Accomplished with difficulty throughout the shift - Did not accomplish - N/A – only one client (skip to Q6) |
|  | **Maelezo / Notes** | |
| 6 | Kuhama toka skrini moja hadi nyingine kwa mteja yule yule (hali ya mama na mtoto tumboni) (🗹moja)   - Imekamilika kwa urahisi tangu mwanzo - Imeendelea kuwa rahisi kukamilika kadiri muda wa kazi ulivyooenda - Imekamilika kwa shida kipindi chote cha zamu - Haikukamilika | Moving between screens for one client (i.e., maternal and fetal well-being)  (🗹 one)   - Accomplished with ease from the start - Accomplished with increasing ease over course of shift - Accomplished with difficulty throughout the shift - Did not accomplish |
|  | **Maelezo / Notes** | |
| **B** | **Majibu ya ePartogram/ Response to the ePartogram** | |
| 7 | Je ilitoa vikumbusho wakati wa zama? (kama kupitilizia, au wakatiwa vipimo) (🗹moja)   - Ndiyo - Hapana (ruka mpaka swali na. 9) | Was a reminder given during the shift? (i.e., “Overdue” or “Due” measurements) (🗹 one)   - Yes - No (skip to 9) |
|  | **Maelezo / Notes** | |
| 8 | Kama ndiyo, je mhudumu alifanya nini?  (🗹 kwa zote zilizo sahihi)   - Alichukua vipimo - Aliidharau - Alihamisha kifaa - Aliuliza ili apate maelezo - Alionekana kuchanganyikiwa na kutojua cha kufanya - Alionekana kufahamu na kuifurahia - Mengineyo ______________________ | If yes, what did the provider do?  (🗹 all that apply)   - Took measurements - Ignored - Moved tablet - Asked for clarification or explanation - Seemed bothered or frustrated - Seemed appreciative - Other __________________________ |
|  | **Maelezo / Notes** | |
| 9 | Je, ilitoa tahadhari wakati wa kuhama? (kama “Alert” au “Attention”)   - Ndiyo - Hapana (ruka mpaka swali na. 11) | Was an alarm given during the shift? (i.e., “Alert” or “Attention”)   - Yes - No (skip to 11) |
|  | **Maelezo / Notes** | |
| 10 | Kama Ndiyo, je mhudumu alifanya nini?  (🗹 kwa zote zilizo sahihi)   - Alichukua vipimo - Aliidharau - Alihamisha kifaa hiki (tablet) mahali pengine - Aliuliza ili apate maelezo - Alitoa huduma zaidi, elezea:   ________________________________   - Alitafuta msaada - Aliomba mgonjwa ahamishwe - Alionekana kuchanganyikiwa na kutojua cha kufanya - Alionekana kufahamu na kuifurahia - Mengineyo _______________________ | If yes, what did the provider do?  (🗹 all that apply)   - Took measurement - Ignored - Moved tablet to another location - Asked for clarification or explanation - Instituted additional care, specify:   _______________________________   - Called for help - Requested transfer for client - Seemed bothered or frustrated - Seemed appreciative - Other _________________________ |
|  | **Maelezo / Notes** | |
| **C** | **Kukubalika / Acceptability** | |
| 11 | Je, SBA alionekana kuwa sawa na kufurahia kutumia ePartogram? (🗹moja)   - Ndiyo – tangu mwanzo - Ndiyo – sawa zaidi jinsi muda wa kazi ulivyoendelea - Hapana – hakuwa sawa wakati wowote | Did SBA seem comfortable using the ePartogram? (🗹 one)   - Yes – from start - Yes – increasingly throughout shift - No – at no point during shift |
|  | **Maelezo / Notes** | |
| 12 | Je, SBA alionekana kujiaminiwa na namna anavyotumia ePartogram? (🗹moja)   - Ndiyo – tangu mwanzo - Ndiyo – zaidi jinsi muda wa kazi ulivyoendelea - Hapana – hakuwa msiri wakati wote kuitumia | Did SBA seem confident using the ePartogram? (🗹 one)   - Yes – from start - Yes – increasingly throughout shift - No – at no point during shift |
|  | **Maelezo / Notes** | |
| 13 | Je, kuingiza taarifa kwenye ePartogram kulifanya utoaji huduma kwa mgonjwa wengine? (🗹moja)   - Ndiyo – tangu mwanzo - Ndiyo – lakini ilipungua kadiri muda wa kazi ulivyoenda - Hapana – haikuchelewesha wakati wowote wa kazi | Did inputting data to the ePartogram delay care for any clients? (🗹 one)   - Yes – from start - Yes – less so throughout shift - No – at no point during shift |
|  | **Maelezo / Notes** | |
| 14 | Je, taarifa za vipimo vya mteja ziliingizwa kwenye ePartogram wakati gani?  (🗹 kwa zote zilizo sahihi)   - Wakati vipimo vinachukuliwa, kila baada ya kipimo - Wakati vipimo vinachukuliwa, kila baada ya vipimo vichache - Mwishoni mwa uchunguzi wa mteja - Wakati mwingine ______________ | When was client data entered into the ePartogram?  (🗹 all that apply)   - As taken, after each measurement - As taken, after every few measurements - At end of the physical exam - Other________________________ |
|  | **Maelezo / Notes** | |
| **D** | **Utunzaji wa tablet / Caring for the tablet** | |
| 15 | Je, betri imekwisha chaji?  (🗹 kwa zote zilizo sahihi)   - Ndiyo – SBA alianza kutumia tableti ya akiba - Ndiyo – SBA akaanza kutumia karatasi/kadi - Ndiyo – SBA aliendelea kutoa huduma bila nyenzo nyingine ya kukusanya taarifa - Ndiyo – SBA alitegemea rekodi za CO - Hapana – betri ilidumu wakati wote wa kazi - Hapana – betri ilichajiwa wakati wa kazi | Did the battery die?  (🗹 all that apply)   - Yes – SBA started using back-up tablet - Yes – SBA started using paper - Yes – SBA continued with care without any other tool - Yes – SBA relied on CO documentation on paper partograph - No – battery lasted entire shift - No – battery charged during shift |
|  | **Maelezo / Notes** | |
| 16 | Je, ni wakati gani tablet iliongezewa umeme au ilichajiwa? (🗹 kwa zote zilizo sahihi)   - Haikuongezewa umeme wakati wote wa kazi - Baada ya kuonyesha onyo kuwa betri inaisha chaji - Mwishoni mwa kazi - “Wakati wa mapumziko”- pale ambapo hamuhudumii mteja - Baada ya betri kuisha chaji - Wakati mwingine _________________ | When was the tablet charged?  (🗹 all that apply)   - Was not charged during shift - After low battery warning - End of shift - “Down time”- when not treating a client - After battery died - Other _________________________ |
|  | **Maelezo / Notes** | |
| 17 | Je, SBA aliihifadhi wapi tablet wakati ambao alikuwa haitumii? (🗹 kwa zote zilizo sahihi)   - Alikuwa nayo mkononi wakati wote - Mfukoni - Mezani, mahali pa kati panapofahamika - Alimpa mfanyakazi mwingine - Aliiweka kwenye chaja (ipate umeme) - Aliweka karibu na chaja (haipati umeme) - Nyinginezo ______________________ | Where did the SBA place/store the tablet when not in use? (🗹 all that apply)   - In hand at all times - In pocket - On table at central location - With another colleague - In charger (charging) - Near charger (not charging) - Other _________________________ |
|  | **Maelezo / Notes** | |
| 18 | Je, SBA aliisafishaje skrini ya tableti?  (🗹 kwa zote zilizo sahihi)   - Aliifuta na pamba kikavu      - Aliifuta na pamba yenye mchanganyiko wa 1:1 wa 70% isopropyl na distilled water - Nyingineyo ______________________ - Hakusafisha tableti | How did the SBA clean the tablet screen?  (🗹 all that apply)   - Dry wipe - Wet wipe – 1:1 ratio of 70% isopropyl and distilled water - Other__________________________ - Did not clean tablet |
|  | **Maelezo / Notes** | |
| 19 | Je, SBA aliisafishaje cover ( kasha la tablet)?  (🗹 kwa zote zilizo sahihi)   - Aliifuta na pamba kikavu - Aliifuta na pamba yenye mchanganyiko 0.1% chlorine solution - Nyingineyo ______________________ - Hakusafisha cover ya tableti | How did the SBA clean the tablet cover?  (🗹 all that apply)   - Dry wipe - Wet wipe – 0.1% chlorine solution - Other _________________________ - Did not clean tablet cover |
|  | **Maelezo / Notes** | |

| **Maswali ya SBA kuhusu ePartogram / SBA questions about the ePartogram** *(to be reviewed daily by study staff)* | **CO aliweza kusaidia? / CO able to help?** |
| --- | --- |
|  | 🞎 Yes 🞎 No |
|  | 🞎 Yes 🞎 No |
|  | 🞎 Yes 🞎 No |
|  | 🞎 Yes 🞎 No |
|  | 🞎 Yes 🞎 No |
|  | 🞎 Yes 🞎 No |
