# Supplementary material for: Use of an electronic Partograph: feasibility and acceptability study in Zanzibar, Tanzania
Source: BMC Pregnancy Childbirth. 2018 May 9;18:147. doi: 10.1186/s12884-018-1760-y (PMC5944152; doi:10.1186/s12884-018-1760-y)
Supplement: Supplementary file 3 — Short Interview Guide: Data Collection Tool 2. Bilingual primary data collection tool that trained study clinical observers used to interview skilled birth attendants after they first five clinical shifts using the ePartogram (DOCX 31 kb) [file 12884_2018_1760_MOESM3_ESM.docx]

**MWONGOZO WA MAHOJIANO MAFUPI NA MKUNGA MTAALAMU / SKILLED BIRTH ATTENDANT (SBA) SHORT INTERVIEW GUIDE**

**Data Collection Tool 2**

*(Itatumiwa na watabibu waangalizi kwa wakunga wataalam kila baada ya wateja watano ambao mkunga alitumia ePartogram)*

*(clinical observer (CO) to administer to SBA following first five clients with whom each SBA uses ePartogram)*

**Study Title:** Feasibility of ePartogram Use in Zanzibar

**Principal Investigador:** Patricia Gomez

**IRB No.:** 6146

**PI Version No./Date:** v2 / 2015 April 22

| SBA study ID |  |  |  |  | | Date | | 2015 |  |  |  |  |
| --- | --- | --- | --- | --- | --- | --- | --- | --- | --- | --- | --- | --- |
|  |  |  |  |  |  |  |  | Year | Month | | Day | |
| Namba ya Mteja. (weka alama ya vyema) | 1🞎 2🞎 3🞎 4🞎 5🞎 | | | | Start time | |  | | End Time | |  | |
| CO Name |  | | | | | | | | | | | |

**Ningependa kutumia dakika kumi na wewe kusikia zaidi kuhusu matumizi yako ya hivi karibuni ya ePartogram. Kama ukumbusho, utaombwa kufanya hivi baada ya wateja wa kwanza 5 ambao utatumia nao ePartogram. Dhumuni la haya mahojiano mafupi ni kupata maoni yako ya kutumia ePartogram ili kufahamisha mabadiliko ya baadae ya muundo, utambulisho na utekelezaji wa ePartogram.**

I would like to spend about ten minutes with you to hear more about your most recent use of the ePartogram. As a reminder, you will be asked to do this after the first five clients with whom you use the ePartogram. The purpose of this short interview is to capture your fresh impressions of using the ePartogram to inform future iterations of the design, introduction and implementation of the ePartogram.

**Acha nikuelezee kidogo kuhusu utaratibu mzima. Nitakuwa nakuuliza maswali kadhaa na kukuomba ujibu kwa uelewa wako mwenyewe. Chukua muda wa kutosha na niulize kama unahitaji ufafanuzi kuhusu nini kinahitajiwa. Tafadhali weka akilini kwamba unaweza kuchagua kuto kujibu swali lolote, na taarifa zote utakazotoa zitakuwa za siri. Unaweza kusitisha mahojiano muda wowote.** **Umeniruhusu kunasa mahojiano yetu?** **[KAMA HAPANA CHUKUA TAARIFA ZA KINA. KAMA NDIO BONYEZA REKODI AU KAMA UNANASA SAUTI TAJA: JINA LA CO, NAMABA YA UTAFITI YA SBA, TAREHE, MUDA WA KUANZA]**

Let me tell you a little about the process. I will be asking you a range of questions and request that you answer questions in your own words. Take your time and ask me to clarify if you have any questions about what is wanted. Please keep in mind that you may choose to not answer any question and that all information you provide will be confidential. You can stop the interview at any time. Do I have your permission to record our interview? [IF NO, TAKE DETAILED NOTES. IF YES, PRESS RECORD AND STATE: CO NAME, SBA STUDY ID, DATE, START TIME.]

1. **Tafadhali nielezee mtazamo wako kwa ujumla wa ePartogram.**

Please tell me your overall impression of using the ePartogram.

1. **Elezea ulifurahia vipi, au la, kutumia ePartogram. *[*DADISI]: Nini ilikuwa rahisi? Nini ilikuwa changamoto? Ilichukua muda mwingi au kidogo ukilinganisha kukamilisha ukilinganisha na partograph ya karatasi? [KAMA SI MTEJA WA KWANZA] Unalinganisha vipi na mara ya mwisho ulivyoitumia?**

Describe how comfortable, or not, you were using the ePartogram. [PROBES]: What was easy? What was challenging? Did it take more or less time to complete compared to a paper partograph? [IF NOT FIRST CLIENT] How did it compare to previous time using it?

1. **ePartogram ina vipengele kadhaa ambavyo havipo kwenye partogtraph ya karatasi. Ningependa kupata mawazo yako kuhusu hivi vipengele. ePartogram ina vikumbusho na vionyo. Vikumbusho vinakukumbusha ni wakati gani vipimo vya mteja vinahitajika, na vionyo hutokea wakati vipimo uliyoingiza havipo kwenye masafa ya kawaida. [DADISI: kama SBA ameelewa tofauti ya Vionyo na vikumbusho]**

I’d like to speak with you about two functions of the ePartogram that a traditional paper partograph cannot offer – alerts and reminders. I’d like to get your thoughts on these functions. The ePartogram has both reminders, which remind you when client measurements are due, and alerts for when a value you have entered is not in the normal range.

**Kwanza kabisa, ningependa kujua unajiskia vipi kuhusu vikumbusho. [DADISI]: Vilikuwa vinasaidia au vilikuwa vinakubugudhi? Vilikuwa vingi sana? Ilikuwa inaeleweka kikumbusho kilikuwa cha nini? Kikumbusho kilikushtua ufanye maamuzi?**

First of all, I would like to know how you felt about the reminders. [PROBES]: Were these helpful or distracting? Were there too many? Was it clear what the reminder was for? Did the reminder prompt you take action?

1. **Sasa, tafadhali nieleze unajisikiaje kuhusu vionyo. [DADISI]: Ilikuwa inaeleweka kwa nini kulikuwa na onyo? Uliona vinasaidia au vinakubugudhi? Uligundua kwamba kuna viwango vya juu na vya chini vya vionyo? Iliathiri maamuzi yako? Ulifanya nini kulivyokuwa na onyo?**

Now, please tell me how you felt about the alerts. [PROBES]: Was it clear why there was an alert? Did you find these distracting or helpful? Did you notice there were both high- and low-level alerts? Did this affect your decisions? What did you do when there was an alert?

1. **[ULIZA KAMA MKUNGA MTAALAMU ALISIMAMIA ZAIDI YA MTEJA MMOJA KWA WAKATI MMOJA AKITUMIA EPARTOGRAM.] Nieleze jinsi ulivyoamua nani wa kumhudumia kwanza ukiwa unatumia ePartogram. [DADISI]: Uliona kama wateja wanapewa kipaumbele kwenye skrini inyoonyesha wateja wote ya ePartogram? Je, kuwapa kipaumbele kulishawishi maamuzi yako kuhusu mpangilio wa kuwahudumia wateja? Ni vigezo gani vingine vilishawishi maamuzi yako? Utaratibu wa kituo? miongozo? Ulielekezwa na mhudumu mwingine? Maamuzi binafsi ya kitabibu?**

[ASK IF THE SBA MANAGED MORE THAN ONE CLIENT AT A TIME USING THE EPARTOGRAM.] Tell me about how you decided who to take care of first when using the ePartogram. [PROBES]: Did you notice clients are prioritized on the home screen of the ePartogram? Did the prioritization influence your decision on the order of which client you cared for? What other factors influenced your decision? Facility norms? SOPs? Instructed by another colleague? Independent clinical judgment?

1. **Kama unavyojua, sio kwamba partogram yote iko kwenye skrini – kuna skrini moja ya kumfuatilia mtoto aliye tumboni na nyingine ya kumfuatilia mzazi. Uliionaje hiyo? [DADISI]: Ilikuwa rahisi zaidi au ngumu zaidi kusoma na kutafsiri takwimu? Ilisaidia au ilileta changamoto katika kufanya maamuzi ya kitabibu?**

As you know, the entire partogram is not on one screen – there is one screen for fetal monitoring and a second for maternal monitoring. How did you find that? [PROBES]: Was it easier or more challenging to read and interpret the data? Did it help or present challenges in making clinical decisions?

1. **Sasa, baada ya kutumia ePartogram wakati ukimhudumia mteja mwenye uchungu, tafadhali nieleze jinsi gani tungefanya wakati wa mafunzo ili uwe tayari zaidi kuitumia.**

Now that you have used the ePartogram while caring for a client in labor, please tell me how we could have better done the training to prepare you.

1. **Tafadhali nieleze kama una mawazo yoyote, maoni au ushauri ya jinsi ya kutambulisha ePartogram kwenye vituo vingine.**

Please tell me any ideas, thoughts or advice you have on how to introduce the ePartogram to other facilities.

1. **Kuna jambo lolote unapenda kunishirikisha?**

Is there anything else you would like to share with me?

**Asante kwa kuchukua muda kujibu haya maswali. [KAMA UNANASA MAJADILIANO ELEZEZA: MUDA WA KUMALIZA]**

Thank you for taking the time to answer these questions. [IF RECORDING STATE: END TIME]
