# Supplementary material for: Use of an electronic Partograph: feasibility and acceptability study in Zanzibar, Tanzania
Source: BMC Pregnancy Childbirth. 2018 May 9;18:147. doi: 10.1186/s12884-018-1760-y (PMC5944152; doi:10.1186/s12884-018-1760-y)
Supplement: Supplementary file 4 — In-Depth Interview Guide: Data Collection Tool 3. Bilingual primary data collection tool that trained study clinical observers used to interview skilled birth attendants at the end of the study period (DOCX 34 kb) [file 12884_2018_1760_MOESM4_ESM.docx]

**MWONGOZO WA MAHOJIANO YA KINA NA WAKUNGA WATAALAMU**

**SKILLED BIRTH ATTENDANT (SBA) IN-DEPTH INTERVIEW GUIDE**

**Data Collection Tool 3**

*(Itatumiwa na watabibu waangalizi kwa wakunga wataalam kila baada kumamaliza matumizi ya ePartogram kwenye kituo)*

*(to be administered to SBA by CO after completion of ePartogram use at facility)*

**Study Title:** Feasibility of ePartogram Use in Zanzibar

**Principal Investigator:** Patricia Gomez

**IRB No.:** 6146

**PI Version No. /Date:** v2 / 2015 April 23

| SBA study ID |  |  | |  | |  | | Date | 2015 |  |  |  |  |
| --- | --- | --- | --- | --- | --- | --- | --- | --- | --- | --- | --- | --- | --- |
|  |  |  |  |  |  |  |  |  | Year | Month | | Day | |
| Start time |  | | End Time | |  | | CO Name | |  | | | | |

**Asante tena kwa kushiriki kwenye hii tafiti katika muda huu wa wiki mbili. Dhumuni la mahojiano ya leo ni kuelewa vizuri uzoefu wako wa kutumia ePartogram kuhudumia wateja wakiwa kwenye uchungu. Kwa vile tuna matumaini ya kutekeleza matumizi ya ePartogram kwenye mifumo kadhaa ya wodi za uzazi, tuna matumaini ya kujifunza mengi kutokana na uzoefu wako wa kutumia ePartogram.**

**Acha nikuelezee kidogo kuhusu utaratibu mzima. Nitakuwa nakuuliza maswali kadhaa na kukuomba ujibu kwa uelewa wako mwenyewe. Chukua muda wa kutosha na niulize kama unahitaji ufafanuzi kuhusu nini kinahitajiwa. Tafadhali weka akilini kwamba unaweza kuchagua kuto kujibu swali lolote na taarifa zote zitakuwa za siri. Unaweza kusitisha mahojiano muda wowote. Maoni yako ni ya muhimu sana. Unaniruhusu kunasa sauti ya mahojiano haya. [KAMA HAPANA ANDIKA MAHOJIANO. KAMA NDIO, ANZA KUNASA SAUTI, KAMA NDIYO ANZA KUNASA SAUTI NA TAJA JINA , NAMABA YA SBA, TAREHE NA MUDA WA KUANZA.]**

Thank you again for participating in this study over the past two weeks. The purpose of today’s interview is to better understand your overall experience using the ePartogram to manage clients in labor. As we are hoping to implement use of the ePartogram in various labor and delivery ward settings, there is a lot we hope to learn from your experience using the ePartogram

Let me tell you a little about the process. I will be asking you a range of questions and request that you answer questions in your own words. Take your time and ask me to clarify if you have any questions about what is wanted. Please keep in mind that you may choose to not answer any question and that all information you provide will be confidential. You can stop the interview at any time. Your opinions are very important. May I have your permission to record this interview? [IF NO, TAKE NOTES. IF YES, START RECORDING AND STATE NAME, SBA NUMBER, DATE, AND START TIME.]

1. **Kwa kuanzia, tafadhali nieleze kwenye zamu ngapi ulitumia ePartogram? ___ zamu**

To begin, please tell me on about how many shifts did you use the ePartogram? ___ shifts

1. **Ni idadi gani ya juu kabisa ya wateja ambao ulitumia nao ePartogram kwa wakati mmoja? ___ wateja kwa wakati mmoja [KAMA “1” NENDA Q4]**

What was the highest number of clients with which you used the ePartogram at one time? ___ patients concurrently [IF “1” SKIP TO Q4]

1. **Nieleze kuhusu hiyo. [DADISI]: Walikuwa wateja wengi sana? Ni idadi gani ya juu kabisa ya wateja uliyohisi nafuu kuwasimamia kwa kutumia ePartogram? Ulihisi ilikuwa rahisi zaidi au ngumu zaidi kuwasimamia idadi hii ya wagonjwa na ePartogram ukilinganisha na partograph ya karatasi?**

Tell me about that. [PROBES]: Was it too many clients? What is the highest number of clients with which you felt comfortable managing using the ePartogram? Did you feel it was easier or more difficult to manage this number of patients with the ePartogram as compared to a paper partograph?

1. **Fafanua jinsi ulivyoibeba, ulivyoisafisha na kuichaji tableti ukiwa kwenye wodi ya uzazi. [DADISI]: je uliibeba tableti toka sehemu moja hadi nyengine kwa kipindi cha kumhudumia mteja? - kama ndivyo wapi ulienda nayo? Je ulijisikia ajabu, au ilikuwa rahisi kuizoea? Ulikuwa unaiweka wapi kati ya kuchukua vipimo? Je, betri ilishawahi kuishiwa chaji wakati unamhudumia mteja? Ni mara ngapi ulikuwa unaisafisha tableti, na nini?**

Describe how you carried, cleaned and charged the tablet in the labor and delivery ward. [PROBES]: Did you take the tablet from one place to another during the shift? Tell me about that. – was it awkward, or was it easy to adjust to? Where did you keep it in between taking measurements? Did the battery ever die while you were managing a client? How often did you clean the tablet, and with what?

1. **Nieleze kuhusu mtazamo wako wa jumla wa ePartogram. [DADISI]: Unaweza kuniambia kitu maalum ambacho umekipenda? Tafadhali niambie kitu chochote ambacho ungependa kibadilishwe au kiboreshwe.**

Tell me about your overall impression of the ePartogram. [PROBES]: Can you tell me something specific that you liked? Please tell me something that you would change or improve.

1. **Sasa, nataka kukuuliza maswali machache kuhusu vipengele vya ePartogram. Vipi kuhusu muonekano wa ePartogram? [DADISI]: Je mwanga wa skrini ulikua unatosheleza? Hata katika masaa tofauti ya siku au maeneo tofauti?**

Now, I’d like to ask you a few questions about the functions and features of the ePartogram. How about the appearance of the ePartogram? [PROBES]: Was the brightness of the screen sufficient, even at different times of day or in different locations?

1. **Je, umeonaje muonekano wa skrini na jinsi takwimu zinavyoingizwa? [DADISI]: Je ilikua rahisi au ngumu kubadilisha skrini kupata taarifa?[VIDOKEZO]: Skrini nne; ya kufuatilia, ya mtoto tumboni, ya mjamzito, na maalezo.**

How about the layout of screens and how data is entered? [PROBES]: How easy or difficult was it to navigate between screens to find data? [HINT]: Four screens – monitor, fetal, maternal, and notes.

1. **Nieleze unafikiria nini thamani ya vikumbusho na vionyo. [DADISI]: Je, viliathiri huduma uliyompa mama wakati wa uchungu? Je, viliongeza thamani gani, kama ilikuwepo, katika kumhudumia mama kipindi cha uchungu?**

Tell me what you think the value of the reminders and alerts is. [PROBES]: Did they affect the care you gave the woman during labor? What value, if any, do they add to taking care of women in labor?

1. **Nieleze jinsi gani unavyo ilinganisha ePartogram na partograph ya karatasi. [DADISI]: Je, inachukua muda mrefu zaidi au mfupi zaidi kukamilisha? Rahisi zaidi au ngumu zaidi kusimamia wateja wengi?**

Tell me how using the ePartogram compares to using the paper partograph. [PROBES]: Does it take more or less time to complete? Is it easier or more difficult to manage multiple clients?

1. **Ulijiskiaje kuhusu vipengele vya ziada vya ePartogram ambavyo havipatikani kwenye partograph . [DADISI]: Vipi kuhusu kipaumbele cha wateja kwenye skrini inayyoonyesha wateja wote? Je, ilikuwa rahisi au vigumu kuzitafsiri grafu??**

How did you feel about the additional functionality the ePartogram has that the paper partograph does not offer.[ PROBES:] How about client prioritization on the home screen? How easy or difficult was it to interpret the graphs?

1. **Unafikiri nini ni tofauti kubwa kabisa kati ya ePartogram na partograph? [DADISI]: Niambie kuhusu faida za kuitumia ePartogram. Na vipi kuhusu hasara zake?**

What do you think is the biggest difference between the paper partograph and the ePartogram? [PROBES]: Tell me about the benefits of using the ePartogram. How about the disadvantages?

1. **Kwa vile tutakuwa tunafanyia kazi toleo la pili la programu-tumizi ya ePartogram, kuna mabadiliko yoyote au marekebisho ambayo unayapendekeza? Kuna kitu chochote ambacho usingependa kibadilishwe?**

As we will be working on a second version of the ePartogram application, are there any changes or modifications that you would recommend? Anything you feel strongly should remain the same?

1. **Kama tutaitambulisha ePartogram kwenye kituo kingine, ni maboresho gani ungependekeza kwa ajili ya mafunzo ya watoa huduma? Kuna kitu chochote ungependekeza kibaki hivyo hivyo?**

If we do introduce the ePartogram in another facility, what improvements would you suggest for training of providers? Is there anything you would suggest stay the same?

1. **Kutokana na uzoefu wako wa kuitumia ePartogram, ungekishauri vipi kituo kingine wakati wa kutoa maamuzi ya kuitambulisha au kutoitambulisha ePartogram kwenye wodi yake ya uzazi? [DADISI]: Nieleze zaidi kuhusu hiyo.**

Based on your experience using the ePartogram, how would you advise another facility deciding whether or not to introduce the ePartogram into its labor and delivery ward? [PROBES]: Tell me your reasons for that decision. Tell me more about that.

**Hayo ndio maswali yote niliyokuwa nayo kwa ajili yako. Asante tena kwa kuchukua muda kuongea na mimi leo. [UKIWA UNANASA SAUTI TAJA MUDA WA KUMALIZA]**

Those are all of the questions I have for you. Thank you again for taking the time to speak with me today. [IF RECORDING, STATE END TIME.]
